# No Isolate, No Problem: Using a Novel Insertion Sequence PCR to Link Rats to Human Shigellosis Cases in an Underserved Urban Community

**DOI:** 10.1128/spectrum.04777-22

**Published:** 2023-05-31

**Authors:** Gordon Ritchie, Victor Leung, Chelsea G. Himsworth, Kaylee A. Byers, Lisa K. F. Lee, Samuel D. Chorlton, Aleksandra Stefanovic, Marc G. Romney, Nancy Matic, Christopher F. Lowe

**Affiliations:** a Division of Medical Microbiology and Virology, St. Paul’s Hospital, Vancouver, British Columbia, Canada; b Department of Pathology and Laboratory Medicine, University British Columbia, Vancouver, British Columbia, Canada; c British Columbia Regional Centre, Canadian Wildlife Health Cooperative, Abbotsford, British Columbia, Canada; d School of Population and Public Health, University of British Columbia, Vancouver, British Columbia, Canada; e Pacific Institute on Pathogens, Pandemics and Society, Simon Fraser University, Burnaby, British Columbia, Canada; f Department of Veterinary Pathology, Western College of Veterinary Medicine, Saskatoon, Saskatchewan, Canada; Institut National de Santé Publique du Québec

**Keywords:** *Rattus norvegicus*, *Shigella flexneri*, insertion sequence

## Abstract

During an investigation into a cluster of Shigella flexneri serotype 2a cases in an underserved community, we assessed the relatedness of human and rat S. flexneri isolates utilizing a novel PCR targeting insertion sites (IS-PCR) of mobile elements in the Shigella genome characteristic of the cluster strain. Whole-genome sequences of S. flexneri (*n* = 50) associated with the cluster were analyzed. *De novo* genome assemblies were analyzed by a Geneious V10.2.6 motif search, and two unique IS were identified in all human *Shigella* sequences of the local cluster. Hydrolysis probe PCR assays were designed to detect these sequences consisting of forward and reverse primers to amplify across each insertion site and a hydrolysis probe spanning the insertion site. IS-PCR was performed for three Shigella PCR-positive culture-negative rat intestine specimens from this community. Both insertion sites were detected in the *de novo* genome assemblies of all clinical S. flexneri isolates (*n* = 50). Two of the three PCR-positive culture-negative rat samples were positive for both unique ISs identified in the human S. flexneri isolates, suggesting that the rat Shigella species strains were closely related to the human strains in the cluster. The cycle threshold (Ct) values were >35, indicating that the bacterial load was very low in the rat samples. Two unique IS were identified in clinical isolates from a community S. flexneri cluster. Both IS targets were identified in PCR-positive (Shigella spp.), culture-negative rat tissue and clinical isolates from humans, indicating relatedness.

**IMPORTANCE** This article describes a novel molecular method to show relatedness between bacterial infections, which may not be able to grow in the laboratory due to treatment with antibiotics or for bacteria requiring unique conditions to grow well. Uniquely, we applied this technique to Shigella isolates from human cases associated with a local cluster in an underserved community, as well as rat samples from the same community. We believe that this novel approach can serve as a complementary method to support outbreak/cluster investigation for Shigella spp.

## OBSERVATION

Shigella spp. have high potential for transmission from person-to-person through fecal-oral contact due to their low infectious dose (10 to 100 organisms) (1). Humans are thought to be the only natural reservoir for Shigella spp., and outbreaks/clusters have been reported in a variety of settings, including daycares/schools, men who have sex with men, foodborne transmission, traveling, and regions with poor hygiene and sanitation (2, 3).

There are numerous methods available for outbreak analysis (pulsed-field gel electrophoresis [PFGE], amplified fragment length polymorphism [AFLP], random amplified polymorphic DNA [RAPD], variable number tandem repeat [VNTR], multilocus sequence typing [MLST], and core genome MLST [cgMLST]), but these methods are dependent on growth of the bacterial isolate in culture (4). Culture-dependent methods are challenging when working with fastidious organisms or samples with low burdens of pathogen. Cases of culture-negative shigellosis have been increasingly recognized due to the use of molecular diagnostics (5, 6), rendering many of these typing methods ineffective. These methods are also resource intensive, requiring specialized instrumentation and technical/bioinformatics expertise. Culture-independent typing methods that are scalable and simple to perform are currently needed to enhance outbreak investigations. Specifically for Shigella spp., which contain a high number of mobile genetic elements and genomic rearrangements, strain relatedness can potentially be analyzed through targeting of insertion sequences (3, 7).

In 2021, a cluster of Shigella flexneri serotype 2a cases occurred in an underserved neighborhood in Vancouver, British Columbia. A specific source (e.g., housing, food, sexual exposures, travel) was not identified, but poor sanitary conditions in this community were presumed to have contributed to ongoing transmission (https://www.vch.ca/en/staff-hub/physicians-and-nurse-practitioners-update). Rats were hypothesized to be one potential vector for ongoing transmission based on previous observations (8). Although samples from rats (Rattus norvegicus) recovered from this community were culture-negative for Shigella spp., three rat samples tested positive for Shigella/enteroinvasive Escherichia coli (EIEC) using PCR (Biofire FilmArray Gastrointestinal Panel) (C. Himsworth, unpub. data). We utilized a novel PCR targeting insertion sequence (IS-PCR) of mobile elements in the Shigella genome characteristic of the outbreak strain for outbreak analysis.

Both insertion sites were detected in the *de novo* genome assemblies of all clinical S. flexneri isolates (*n* = 50), which represented samples from the course of the outbreak. Two of the three PCR-positive culture-negative rat samples were positive for both unique ISs identified in the human S. flexneri isolates, suggesting that the rat Shigella species strains were closely related to the human strains in the outbreak. The cycle threshold (Ct) values for the PCR were >35, indicating that the bacterial load was very low in the rat samples (Table 1). The seven PCR-negative culture-negative rat samples were negative for both unique ISs.

**TABLE 1:** Cycle threshold values from direct amplification of rat intestines^*a*^

Rat	Cycle threshold value		
	*ipaH*	IS-PCR1	IS-PCR2
1	33	35	41
2	36	Negative	Negative
3	29	35	46

a The table shows the cycle threshold values from direct amplification of rat intestines associated with a community S. flexneri outbreak for *ipaH* and two unique insertion sequences (IS-PCR1 and IS-PCR2). IS-PCR, PCR targeting insertion site.

The agilent gel of IS-PCR products gave the expected amplicon sizes from the WGS of the human S. flexneri isolates: 206 and 212 bp. Consensus sequence reads from the positive rat samples were 100% identical to the human isolate sequences, confirming the presence of the IS in the rat samples.

We used a novel strain typing method (IS-PCR) to show that Shigella spp. from humans and rats in the same community were related. In Shigella spp., much of the strain-to-strain differences are due to transposase activity within mobile elements, which result in genome rearrangement. This method uses the insertion site of mobile elements in the Shigella genome to link bacterial strains, as insertion sites are generally stable through the course of an outbreak (3). Two mobile element insertion sites were found in all the human Shigella outbreak isolates that were rare in GenBank and were also detected in the rat samples by amplifying across the insertion site, with enhanced discriminatory power via a hydrolysis probe spanning the insertion site. In addition, gel electrophoresis showed identical sized amplicons for the rat samples and the human isolates, and PCR products were sequenced and matched the targeted IS. The discovery of identical rare insertion sites for IS1 in the human isolates and the rat samples demonstrates that the Shigella strains are related. Although IS-PCR can infer relatedness, it cannot suggest directionality of transmission. In addition, as rats are coprophagic, the detection of low level S. flexneri DNA in rat intestines may be secondary to contact or consumption of Shigella-contaminated food or objects rather than Shigella infection in the rats. Despite this, identification of the human strain in rats can provide public health officers with a potential source to target in mitigating ongoing transmission, although further research is required to understand the role rats may play in the spread of human-associated pathogens such as Shigella or, conversely, transmission of Shigella from humans to rats.

The utilization of IS-PCR enabled typing of culture-negative rat samples. There is a need for culture-independent modalities to support outbreak investigations. Stool cultures may not always be able to isolate Shigella spp. due to antibiotic treatment or nonviable bacteria. With the evolving use of molecular testing for the detection of gastrointestinal pathogens, an isolate may not be readily available for culture-dependent typing methods. PCR-based methods enable typing of samples with low organism burden, which may be below the limit of detection for culture or metagenomics (9). A potential confounding factor at late Ct values is the detection of artifact, but confirmation by sequencing, as done in this study, mitigates this risk. This methodology may also be amenable to outbreak/cluster investigations for other microorganisms, such as Mycobacteria spp., Corynebacterium spp., or Staphylococcus spp., in which strain diversity is similarly achieved through mobile elements traversing throughout the genome (10).

Unique to this study was the detection of Shigella spp. directly from rat intestine and evidence that it shared the same two IS sites as the human outbreak strains. A PCR-based approach was able to identify the IS sites at low levels from nonsterile tissue. This may not be possible with metagenomic sequencing, as other more abundant gastrointestinal flora would be preferentially sequenced, precluding recovery of complete Shigella spp. genomes.

This study is limited by sample size. We applied this novel IS-PCR to only one known outbreak to date, and a potential limitation of the IS-PCR method would be the ability to detect unique insertion sites for each outbreak, which would require WGS of the index outbreak strains. A combination of IS-PCRs for rare insertion sites should also be considered, where the presence of a particular combination of two to three insertion sites may be uniquely identified per outbreak with good discriminatory power. Further work is also needed to assess the specificity of the IS-PCR, including testing of historical samples within our geographical region. As ISs are transposable elements, there is a possibility for movement of the ISs over time. For acute outbreaks over a defined time, it would be unlikely to affect the IS-PCR design but reaffirms the need to reassess the appropriateness of the IS-PCR for subsequent outbreaks. With respect to sensitivity, while the IS-PCR detected the targets at low levels (Ct > 35) in some rats, in cases where the IS-PCR was negative, there is a potential for that to be a false-negative.

In conclusion, IS-PCR is a novel rapid method for determining relatedness of Shigella strains, which we utilized to link clinical human cases to rats. As a PCR-based approach, it may be better suited for direct PCR on tissue or samples where bacterial culture is not possible and can serve as a complementary modality to existing laboratory methods such as WGS for cluster/outbreak investigation.

S. flexneri (*n* = 50) isolates from the outbreak underwent whole-genome sequencing (WGS) using the GridION (GenBank accession number PRJNA947612). All genomes were serotype 2a (*in silico* serotyping with ShigEiFinder) and MLST 245 (15-allele alternate E. coli typing scheme: ST15 sequence type, 100; clonal group, 10) (3, 11). Human S. flexneri isolates associated with the community outbreak were grown overnight in Mueller-Hinton broth and then inactivated at 99°C for 10 min. A total of 250 μL bacterial lysis buffer (Roche) was added to 250 μL bacterial suspension, followed by the addition of 25 μL of proteinase K (Sigma). The suspension was incubated at 65°C for 1 h, and then DNA was extracted on the MagNA Pure 24 (Roche Molecular Systems). The DNA was prepared for sequencing with the SQK-LSK109 ligation sequencing kit with NBD104/114 barcoding. The DNA library was run on GridIon (Oxford Nanopore Technologies) with R9 flowcells. Raw sequencing data were basecalled with Guppy (version 4.1.0) and uploaded to BugSeq for further automated analysis (12). The reads were assembled with metaFlye and polished with Racon, Medaka (https://github.com/nanoporetech/medaka), and Homopolish (13–15). A consensus FASTA sequence was constructed using bugseq.com, and 109 copies of the IS1 mobile element were found in the resulting *de novo* genome assemblies by a Geneious version 10.2.6 motif search. Sequences spanning these insertion sites were scanned by BLAST searches of GenBank (performed August 2022; at the time, >10,000 S. flexneri and >170,000 E. coli genome sequences). One unique insertion site was present in all human Shigella sequences in the local outbreak, but none were present in any other E. coli/Shigella sequences in GenBank (i.e., all publicly available E. coli/Shigella sequences) at the time of primer design. Another rare insertion site was identified that was present in only 40 S. flexneri sequences within GenBank. This site was the result of a fusion of the *acrA*-like gene with the *yegE* gene interrupted by an ISSfl2 insertion sequence. Neither IS was identified in any other ST245 strain in GenBank. A BLAST search for other gastrointestinal pathogens was conducted but did not identify any matches with the IS-PCR products.

Rats were collected from March 23 to April 9, 2021, from six city blocks in Vancouver’s urban downtown eastside neighborhoods. City blocks were selected with highest concentration of positive human S. flexneri cases. We used lethal Snap-e rat traps (Kness Manufacturing Co., Albia, IA) inserted inside tempered PROTECTA EVO Express Bait Stations (Bell Laboratories, Windsor, WI). Six traps were placed in the alleyway of each city block for a total of 36 active traps. We checked the traps each morning, and for the trapped rats, we recorded capture date and location. Following capture, the rats were stored at −20°C prior to undergoing a full necropsy. To collect samples for Shigella testing, we collected each rat’s whole intestine. This was stored at −80°C prior to Shigella spp. testing. Ten rat specimens (intestine) from this community were recovered for IS-PCR (three PCR-positive culture-negative). The rat intestine samples were suspended in 700 μL of phosphate-buffered saline (PBS) and bead lysed with 0.2-mm beads on the Qiagen TissueLyser LT for 3 min, then extracted on the Roche MagNA pure compact, and eluted in 50 μL. To increase the specificity of the PCR, hydrolysis probe PCR assays were designed to detect these sequences consisting of forward and reverse primers to amplify across each insertion site and a hydrolysis probe spanning the insertion site (Table 2). PCR (5 μL DNA) was performed on the Lightcycler 480 (Roche Diagnostics, Laval, Quebec, Canada) using the IDT gene expression master mix (10 μL) with 0.2 μM primer, 0.1 μM probe, and 1 mg/mL bovine serum albumin (BSA). The cycling conditions were 95°C for 10 min, 60 cycles of 97°C for 5 s, 66°C stepping down to 60°C at 0.2°C/cycle for 20 s, and 72°C for 2 s, with fluorescence determination at 510 nm. All primers and probes were synthesized by Integrated DNA Technologies. PCR-positive control was a 1/1,000 dilution of one of the human outbreak isolates. PCR-negative control was PBS that was processed at the same time as the rat samples, including bead lysing and extraction. Bioanalyzer 2100 gel electrophoresis (Agilent Technologies, Santa Clara, CA) was performed on the PCR products. PCR products were barcoded, normalized, and applied to the MinION (R9.4 FLO-MIN106D) according to the Oxford Nanopore 1D Native barcoding protocol (version: NBE_9065_v109_revE_23May2018). All DNA clean-ups were performed with 2× SPRI Agencourt beads to ensure adequate DNA recovery from the low-molecular-weight PCR products. Consensus sequence reads were analyzed with Geneious 10.2.6 software. This study was approved by the University of British Columbia Animal Care Committee (A20-0212) and adhered to guidelines outlined by the Canadian Council on Animal Care.

**TABLE 2:** Primer and probe sequences for the insertion sequence PCR (IS-PCR) and *ipaH* PCR

Primer	Sequence	Amplicon size
IS-PCR1		206 bp
F	GCC ACG CAA GCA CCT TAA AA	
R	ACC GAA AAG CAT CAC CAC CA	
Probe	FAM-CGA TTA TTA ATG GCG GGT AAT GCT GC-BHQ2	
IS-PCR2		212 bp
F	ACT TTG AAC TGG CTG GGG TC	
R	CGC TTG AGC TTT CGC AGT TT	
Probe	FAM-ATA TCG CCG-ZEN-CAT CCA TTC CAG GAA GG-IABKFQ	
*ipaH*-PCR		
F	CCT TTT CCG CGT TCC TTG A	
R	CGG AAT CCG GAG GTA TTG C	
Probe	FAM-CGC CTT TCC-ZEN-GAT ACC GTC TCT GCA-IABKFQ	
